# 2020 and now: what has been accomplished in blindness prevention and what is next?

**DOI:** 10.5935/0004-2749.20200101

**Published:** 2024-02-11

**Authors:** João M. Furtado, Túlio Frade Reis, Kristen A. Eckert, Van C. Lansingh

**Affiliations:** 1 Faculdade de Medicina de Ribeirão Preto, Universidade de São Paulo, Ribeirão Preto, SP, Brazil; 2 Independent consultant, San Antonio Tlayacapan, Jalisco, Mexico; 3 Helpmesee; 4 Instituto Mexicano de Oftalmología, Querétaro, Mexico

## INTRODUCTION TO VISION 2020: THE RIGHT TO SIGHT

In 1995, the World Health Organization (WHO) estimated that, globally, there were 110 million people with visual impairment and 38 million people with blindness, with the number projected to increase to 76 million by 2020^(1,2)^. More concerning was that these numbers excluded uncorrected refractive error which means that the burden of vision loss was very likely higher.

Ninety percent of the global population with blindness lived in developing countries, where people were 10 times more likely to go blind^(3)^. Eighty percent of blindness was avoidable, with 70% at the time being caused by cataract, childhood eye disease, trachoma, and onchocerciasis^(3)^. In response, in 1999, the International Agency for the Prevention of Blindness (IAPB) and WHO launched a global advocacy program, VISION 2020: the Right to Sight (V2020), which aimed to reduce the projected number of the global population with blindness from 75 million to 25 million and eliminate avoidable blindness by 2020^(3,4)^. Governments and health ministries, nongovernmental organizations, industry, every supranational society of ophthalmology, and many national societies of ophthalmology have supported V2020 and the implementation of sustainable eye care programs that provide equitable eye health services to the world’s most vulnerable communities. The program is grounded on 3 core pillars of disease control; human resource development, infrastructure development, and integration with primary health care. V2020 has been overseen by a national blindness prevention or V2020 committee in each country^(4,5)^. It has been estimated that reducing global blindness by 66% by 2020 would prevent 429 million people with blindness-years and would result in a $102 billion gain for the global economy^(1,6)^.

In the 20 years since the inception of V2020, more than 100 national eye care plans have been drafted, more than 100 national V2020 committees have been formed, and great strides have been made to strengthen eye health systems through best-practice eye care models^(1,5)^. However, the success of the scale-up of eye care models, the government financing of the implementation of national plans, the long-term sustainability of national committees, and the integration of eye health into health systems at large, remains to be seen^(1,7)^. On the epidemiological front, the global trend towards increasing vision loss has seen a reverse, and the control of infectious blinding diseases has made remarkable progress^(1)^. There have been four World Health Assembly resolutions passed by WHO Member States since the inception of V2020: WHA56.26 in 2003, WHA59.25 in 2006, WHA62.1 in 2009, and WHA66.11 in 2013^(8)^. The last resolution was embodied in the report, *Universal Eye Health: a global action plan 2014-2019*, which redefined the V2020 goal to eliminate avoidable blindness by aiming for a 25% reduction in avoidable vision loss by 2019, based on the 2010 baseline^(8,9)^. The accomplishments of V2020 and the WHO global action plan and their challenges are discussed in further detail below.

### Accomplishments of V2020

Multiple accomplishments were recorded during the period, as follows:

1. The global prevalence of blindness is declining:
  The global age-standardized prevalence of blindness decreased from 0.75% in 1990 to 0.48% in 2015. A similar trend occurred with the prevalence of moderate and severe visual impairment, which decreased from 3.83% to 2.95%. In 2015 there were 36 million people with blindness and 216.6 million people with moderate and severe visual impairment. The number of blind individuals, however, has increased, mostly due to the increase in the total population and growing life expectancy. The decline of the global prevalence of blindness is in a large part due to the higher number of cataract surgeries being performed^(10)^.
2. The reduction of the contribution of infectious diseases to visual impairment and blindness
  Trachoma and onchocerciasis no longer cause as much blindness as they did when V2020 was launched. Blinding trachoma dropped from 0.9 million cases in 1990 to 0.4 million cases in 2015^(11)^, and the mass distribution of ivermectin for onchocerciasis in Africa is a success story in blindness prevention^(12)^.
3. More and better data on indicators for blindness prevention
  WHO requires three indicsators on blindness prevention from member states: 1) The prevalence and causes of visual impairment and blindness, extracted from population-based studies; 2) The number of eye care personnel by cadre; 3) Cataract surgical service delivery (cataract surgical rate, CSR, and cataract surgical coverage, CSC)^(13)^.

Until the early nineties, little was known about what was causing blindness in vast regions of the world such as Latin America^(14)^, leading to inaccurate estimations and an inability to develop action plans for blindness prevention. Over the last few decades, the number of population-based studies on vision impairment, blindness, and its causes has increased, and this leads to better planning of initiatives. The “Rapid Assessment” methodologies, composed of the Rapid Assessment of Cataract Surgical Services (RACSS) and its evolution, the

Rapid Assessment of Avoidable Blindness (RAAB) are one example. We had 22 studies conducted in the 90s, 112 studies performed in the first decade of the 21^st^ century, and 187 studies conducted from 2010 to 2019^(15)^. Other important studies using non-RAAB methodologies were conducted, such as the Los Angeles Latino Eye Study (LALES)^(16)^, the São Paulo Eye Study and the Brazilian Amazon Region Eye Survey (BARES)^(17)^ among others.

In the last decade, the RAAB methodology incorporated a diabetic retinopathy module, designed for regions where investigators expect a higher prevalence of diabetes. More recently, an app was designed for mobile data collection and the upcoming version of the software will be able to perform visual acuity testing and the incorporation of a low-cost camera for anterior and posterior segment imaging is on the horizon^(15)^.

Not only do we have more studies, but data is more readily available, since there is an increasing effort to publish these studies as scientific manuscripts, and the RAAB Repository was created to provide general information on executed RAAB studies, the principal investigators and their contact information^(15)^.

Until 2010, global estimates generated by WHO considered only “Rapid” studies from a limited number of countries, which limited the accuracy of their estimates^(18)^. The Vision Loss Expert Group (VLEG), an international group created to generate global and regional estimates on blindness, visual impairment, and its causes, published its first estimates in 2013^(19)^, and an update in 2017^(10)^ VLEG also assists the Global Burden of Disease study and WHO in generating data on disease burden, measured by disability-adjusted life years due to vision loss^(20)^.

Since the number of human resources is a limitation in many regions, having an accurate estimate of eye care professionals is paramount. Although, there are challenges, especially in calculating the number of non-ophthalmologists, as a result of the efforts of the In ternational Council of Ophthalmology there is now a better understanding of the magnitude of the global population of ophthalmologists^(21)^.

Having more RAAB studies has resulted in more data on CSC, which is a sub-output of the study. The CSR, defined as the annual number of cataract surgeries performed per year in a given location^(22)^, has been reported over the last fifteen years in many regions such as Latin America^(23)^, although there are issues regarding reliability and consistency in data collection between countries.

More recently, another indicator, the effective cataract surgical coverage (eCSC) was devised by Ramke et al.^(24)^, and it combines cataract surgical coverage with surgical outcomes.

4) Availability of data for lay audience and policymakers
  Not only do we have more and better data, but the numbers, estimates and graphic representations are available in appropriate languages for policymakers, non-governmental organizations and the general public. IAPB launched the Vision Atlas in 2016^(25)^, and recently WHO released the World Report on Vision^(9)^, both aiming to spread information on eye health for non-eye health professionals.
5) Changes in blindness and visual impairment definitions over time
  Until 2006, vision impairment and blindness definition used the term “best-corrected visual acuity”. It means that uncorrected refractive errors were not taken into consideration, and the existing burden was therefore unknown. By modifying it to “presenting”^(26)^ visual acuity (VA), which is the measurement of visual acuity with the optical correction in use (if any), studies started to take into account uncorrected refractive errors, now recognized as the leading global cause of vision impairment, and actions targeting refractive services and spectacle provision were boosted. Recently, the definition was modified again^(27)^ with the addition of a new category (mild vision impairment: 20/40 <VA ≤20/60), and the important inclusion of “near vision impairment”, leading to better estimates of near vision impairment and actions to overcome it.

### Challenges

Besides the multiple achievements mentioned above, there are many hurdles to overcome in blindness prevention, some are listed below:

- Population aging
  As the global population gets older and larger, more people are affected by the commonest causes of vision loss. In 2020 cataract is still estimated to be the main cause of blindness, affecting 13.4 million people, followed by uncorrected refractive error (8 million), glaucoma (3.2 million), and age-related macular degeneration (2 million). The first two causes can be satisfactorily treated by surgery and spectacle correction, respectively. The others and diabetic retinopathy are chronic conditions that demand longer treatment and medical follow-up. Each country has its difficulties to overcome to improve eye health. However, as cataracts are increasingly operated on and refractive errors are corrected, the chronic conditions will gain more importance epidemiologically. In the coming years, countries should develop strategies to fight these conditions^(10,11)^.
- Obesity/diabetes
  The incidence of diabetes is increasing and will affect the projections of the number of people who will be living with diabetic retinopathy and its impact on blindness and visual impairment. Strategies targeting the diagnosis, patient awareness, and compliance, and ocular interventions should be implemented to prevent diabetic retinopathy and the resultant blindness^(28)^.
- Distribution of services, quality, and inequities
  As with many other areas of health, women are less likely to get access to cataract surgical services in many areas of the globe^(29)^. Also, poverty is associated with reduced access and lower quality of ophthalmic services. Poor people tend to have limited access to eye care services, and when they do have access the quality is often below the accepted minimum^(30)^. Reports show that blindness is more common^(31)^ and surgical results are worse in lower-income settings within the same studied country^(32)^.
- Distribution of eye care providers
  Although Resnikoff et al. estimated that there is a growing population of ophthalmologists globally (232,866 in 2015, a 14% increase since 2010)^(21)^, they are more concentrated in high-income countries and in countries where the prevalence of blindness is lower than those with a smaller concentration of professionals. A higher concentration of ophthalmologists in wealthier areas was also found when multiple Latin American countries were analyzed^(33)^.
- An increasing number of people are blind and visua lly impaired despite the decreasing prevalence
  From 1990 to 2015, despite a 0.27% reduction in the prevalence of blindness globally, the number of blind people increased by 5.4 million. It is estimated that in 2050 there will be 114.6 million blind people and 587.6 million with moderate and severe visual impairment^(10)^. Consequently, there will be a higher demand for refractive services, cataract surgical services and the need for the treatment of chronic conditions and rehabilitation services will increase substantially.
- More population-based data is needed
  Although there is a growing number of population-based studies on blindness globally, it is imperative to generate data on locations where there have so far been no studies, to reduce uncertainties in our estimates. As an example, only recently was pterygium identified as an important cause of visual impairment in the Brazilian Amazon region^(31,34)^, whereas previous Brazilian studies, conducted in wealthier areas, did not identify it as a public health issue^(35-37)^.

Also, in regions where the studies were executed more than a decade ago, current data are needed to evaluate changes in patterns and define new strategies. In addition, there is little data on near vision impairment^(38)^, which was only recently included in the WHO definition of visual impairment/blindness^(27)^.

## CONCLUSION

Although the global prevalence of blindness is decreasing, treatable conditions such as cataract and uncorrected refractive errors are still the leading global causes of blindness and visual impairment, respectively^(11)^. It is imperative that strategies to integrate eye care into existing health systems are developed, to increase coverage and reduce gaps. Although many strides have been made in blindness prevention since the inception of V2020, a world free of avoidable blindness is a dream still far from being achieved.
